# Chrononutrition and Polyphenols: Roles and Diseases

**DOI:** 10.3390/nu11112602

**Published:** 2019-10-30

**Authors:** Anna Arola-Arnal, Álvaro Cruz-Carrión, Cristina Torres-Fuentes, Javier Ávila-Román, Gerard Aragonès, Miquel Mulero, Francisca Isabel Bravo, Begoña Muguerza, Lluís Arola, Manuel Suárez

**Affiliations:** 1 Nutrigenomics Research Group, Departament de Bioquímica i Biotecnología, Universitat Rovira i Virgili, 43007 Tarragona, Spain; anna.arola@urv.cat (A.A.-A.); alvarojavier.cruz@urv.cat (Á.C.-C.); cristina.torres@urv.cat (C.T.-F.); franciscojavier.avila@urv.cat (J.Á.-R.); gerard.aragones@urv.cat (G.A.); miquel.mulero@urv.cat (M.M.); franciscaisabel.bravo@urv.cat (F.I.B.); begona.muguerza@urv.cat (B.M.); lluis.arola@urv.cat (L.A.); 2 Technological Unit of Nutrition and Health, EURECAT-Technology Centre of Catalonia, 43204 Reus, Spain

**Keywords:** chrononutrition, biological rhythms, polyphenols, health benefits, diseases, metabolic syndrome, nutrition

## Abstract

Biological rhythms can influence the activity of bioactive compounds, and at the same time, the intake of these compounds can modulate biological rhythms. In this context, chrononutrition has appeared as a research field centered on the study of the interactions among biological rhythms, nutrition, and metabolism. This review summarizes the role of phenolic compounds in the modulation of biological rhythms, focusing on their effects in the treatment or prevention of chronic diseases. Heterotrophs are able to sense chemical cues mediated by phytochemicals such as phenolic compounds, promoting their adaptation to environmental conditions. This is called xenohormesis. Hence, the consumption of fruits and vegetables rich in phenolic compounds exerts several health benefits, mainly attributed to the product of their metabolism. However, the profile of phenolic compounds present in plants differs among species and is highly variable depending on agricultural and technological factors. In this sense, the seasonal consumption of polyphenol-rich fruits could induce important changes in the regulation of physiology and metabolism due to the particular phenolic profile that the fruits contain. This fact highlights the need for studies that evaluate the impact of these specific phenolic profiles on health to establish more accurate dietary recommendations.

## 1. Introduction

### 1.1. Biological Rhythms (Circadian and Seasonal)

Biological rhythms, which are present in all organisms and include circadian and circannual rhythms, are closely related to metabolism and nutrition. In this framework, light plays a pivotal role in relevant changes in the physiological and metabolic signaling pathways that drive the behavior of organisms [1,2]. Moreover, light modulates seasonal-specific behaviors such as reproduction, migration, hibernation, germination or blossom according to the period and organism [3,4,5]. The presence of endogenous rhythms has clearly been an advantage in evolution beginning with cyanobacteria, which represent one of the earliest and most primitive organisms that presents these types of regulatory processes [6]. Nevertheless, although it is known that these reactions are highly regulated by light-induced gene expression, the mechanisms involved are still not clearly identified. In this sense, there is growing evidence that factors affecting the biological clock, such as gene polymorphisms of the core clock machinery and seasonal changes in the light–dark cycle, exert a marked influence on physiological activity.

In recent years, it has been observed that biological rhythm can influence the biological activity of bioactive compounds or nutrients from the diet. Moreover, the intake of nutrients can also modulate biological rhythms. Based on this concept, some authors have reported that the first meal after long-term fasting, such as breakfast, is important for the synchronization of the clock [7]. Furthermore, it is worth highlighting that bioactive compounds such as polyphenols from the diet can also interact with the clock, establishing an accurate time-point for their consumption according to the active signaling pathways [8]. Recently, a link between circadian rhythms, the microbiome, and obesity has been reported in which the gut microbiome is understood as an endocrine system that, linked with circadian rhythms, may influence obesity [9]. In addition, the consumption of functional foods has been shown to have beneficial effects by preventing or ameliorating many chronic physiological diseases, such as obesity and associated metabolic syndrome, at least in part through the modulation of circadian rhythms [10]. All these findings show the importance of circadian rhythms in the physiological and metabolic response of our body and how we can use this knowledge to prevent or ameliorate pathologies such as metabolic syndrome.

### 1.2. Molecular Mechanism

**Figure 1:**
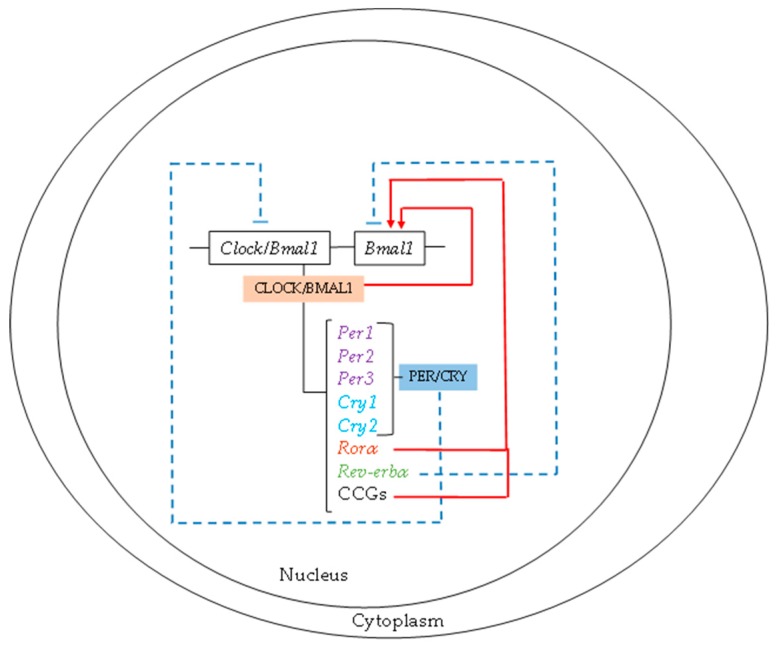
Molecular mechanisms of biological rhythms. The circadian locomotor output cycles kaput/brain and muscle ARNT-like protein 1 (CLOCK/BMAL1) heterodimer is the central clock in all cells and stimulates the transcriptional activity of the period (*Per*) and cryptochrome (*Cry*) genes, whose heterodimer acts as a negative feedback loop of *Clock/Bmal1* transcriptional expression. The two feedback loops of *Rorα* and *Rev-erbα* expression are regulated by CLOCK/BMAL1.

Circadian rhythms ensure that all processes present maximum and minimum values of functionality throughout the day. Currently, it is accepted that the molecular mechanism that drives biological rhythms is composed of a set of two interlocking transcriptional–translational feed-back loops, circadian locomotor output cycles kaput (CLOCK) and brain and muscle ARNT-like protein 1 (BMAL1). The CLOCK/BMAL1 heterodimer is the common central clock in all cells and stimulates the transcriptional activity of three period (*Per*) and two cryptochrome (*Cry*) genes [9]. The PER/CRY heterodimer acts as a negative *feedback loop* of *Clock/Bmal1* transcriptional expression [11]. Moreover, the CLOCK/BMAL1 heterodimer activates *Bmal1* gene expression. Additionally, there are two *feedback loops, Rorα* and *Rev-erbα*, whose expressions are regulated by CLOCK/BMAL1. RORα and REV-ERBα act as activators and inhibitors of *Bmal1,* respectively (Figure 1).

**Figure 2:**
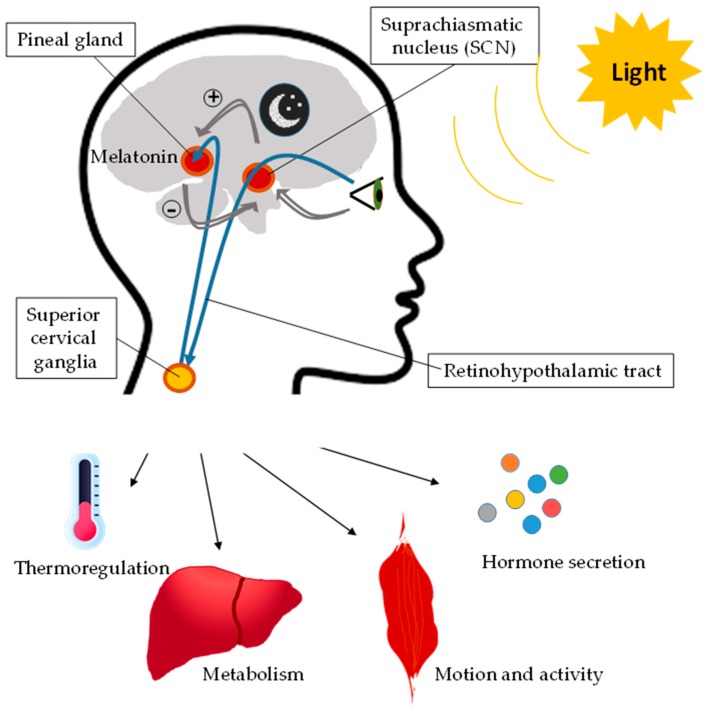
Effect of light on biological rhythms. Light regulates biological rhythms through the activation of the suprachiasmatic nucleus, which actives superior cervical ganglia, which triggers different signaling pathways in the body, such as pathways that modulate thermoregulation, metabolism, motion, activity, and hormone secretion. In the dark period, the pineal gland synthesizes melatonin, which inhibits the action of the suprachiasmatic nucleus.

On the other hand, the CLOCK/BMAL1 *loop* induces the transcription of clock-controlled genes (CCG). The activity of this *loop* determines the circadian rhythm expression of these CCGs and, therefore, the metabolic and functional cellular rhythms. In addition, there is an epigenetic *loop*, which involves sirtuin 1 (SIRT1), nicotinamide phosphoribosyltransferase (Nampt), and nicotinamide adenine dinucleotide (NAD^+^). *Nampt,* a CCG gene overexpressed by CLOCK/BMAL1, increases the intracellular concentration of NAD^+^ with the consequent activation of SIRT1, which induces the deacetylation of BMAL1 and histones, suppressing the transcriptional activity of CLOCK/BMAL1. In addition to NAD, other major intracellular biochemical components with a clock regulator function are monophosphate-activated protein kinase (AMPK) and cyclic adenosine monophosphate (cAMP). There are specific CCGs according to the cell type [12]. Thus, the molecular clock controls cellular functionality and metabolism via CCGs through metabolic pathways or transcriptional factors and nuclear receptors, which induce the expression of enzymes and other metabolic factors [13,14]. Furthermore, light regulates all these processes, triggering the activation of different signaling pathways in the body (Figure 2). In this regard, melatonin plays a pivotal role during the dark cycle, turning off the light-cycle metabolism.

It is well established that mutants in *Clock* and *Bmal1* result in an abnormal metabolic phenotype, characterized by obesity, metabolic syndrome, and type 2 diabetes. These disorders occur due to hypoglycemia and elevated glucose clearance after acute glucose administration in the pancreas [15], hyperglycemia and reduced glucose tolerance in the pancreas [16], decreased insulin-dependent glucose metabolism in the skeletal muscle [17], and abnormal polyunsaturated fatty acid secretion from adipocytes [18], regulating hypothalamic appetite centers in white adipose tissue-specific clock disruption. In recent years, our group has studied circannual rhythms in metabolic homeostasis by analyzing seasonal fluctuations in glucose and lipid metabolism [19] as well as several comorbidities related to obesity, including hypertension and insulin resistance. Our studies focused on how biological rhythms can modulate these pathological parameters by regulating the signaling pathways associated with the circadian rhythm-related genes *Cry1*, *Bmal1*, *Per2*, and *Nr1d1* [20]. The present review summarizes the role of polyphenols in the modulation of biological rhythms, with potential focus on the treatment or prevention of chronic diseases such as metabolic syndrome.

## 2. Chrononutrition

### 2.1. Definition

In the last few years, there has been increasing recognition of the impact of the biological clock on nutrition, with different effects on energy balance and metabolism and influences on health and diseases. This concept has led to the development of a new discipline known as chrononutrition [21], which was first mentioned in a Japanese book about nutrition and health published in 2005 [22]. Since then, chrononutrition has emerged as a research field focused on the study of the interactions between biological rhythms, nutrition, and metabolism [21]. Specifically, chrononutrition involves not only how the timing of food intake and biological rhythms may affect health, metabolism, and nutrition but also on how nutrition (composition and size of meal) may affect our internal clock system [23].

The majority of the clinical studies carried out in this field have mainly focused on the effects of meal timing. In this regard, it has been observed that meal timing patterns such as skipping breakfast, consuming higher-energy meals in the evening, and greater eating and snack frequency are linked with a higher risk of being overweight or obese and with adverse metabolic effects in humans [24]. In fact, changes in circadian eating patterns result in increased energy intake in both humans and rodents [25,26,27,28,29,30]. Moreover, differential effects on weight loss have been shown after meal-timing variations in overweight and obese women [31]. On the other hand, short sleep duration has been associated with the development of different chronic diseases such as hypertension, type 2 diabetes, and obesity [32,33,34]. The mechanisms underlying these effects are not yet fully understood. However, in recent years, various studies have provided new insights. Circadian rhythm alterations lead to increased glucose and insulin levels as well as increased arterial pressure, decreased leptin levels, sleep deficiency, and altered cortisol secretion rhythm [35]. Leptin signaling, which modulates satiety, exhibits circadian variation and may link clock gene fluctuations with metabolic diseases such as diabetes and obesity [35]. Indeed, unusual food intake timing has been linked to altered satiety signals and reduced serum leptin levels [25,36,37]. This reduction in leptin levels leads to increased appetite while reducing energy expenditure and therefore promotes the development of obesity and other metabolic disorders [35]. Another factor that may explain the influence of circadian clocks on metabolism modulation is the effects on thermogenesis and energy expenditure [25]. Thus, thermogenesis is induced by meal intake and follows a circadian rhythm, being highest in the morning, followed by the afternoon and night [25]. Hence, this may explain why skipping breakfast is linked to increased body weight. Recently, it has been shown that this increased diet-induced thermogenesis in the morning may be due to the circadian rhythmicity of circulating norepinephrine and epinephrine, with increased values in the morning; these compounds are known to influence food intake modulation [38,39]. Moreover, in a study of healthy individuals, the same meal consumed in the evening resulted in a lower resting metabolic rate (RMR) and increased glycemic/insulinemic response, suggesting circadian variations in energy expenditure and metabolic pattern [40]. The hunger hormone ghrelin has also shown circadian oscillation, with the lowest levels in the morning, and modulates energy expenditure and thermogenesis via the suppression of brown fat thermogenesis [38].

### 2.2. Macronutrients and Bioactives

On the other hand, as mentioned above, dietary patterns may affect our biological rhythms [23,41]. Hence, the expression levels of clock genes, such as *Per1*/*Per2*, showed variations in the liver with daytime feeding [42,43]. Moreover, the absence of feeding rhythmicity together with the absence of adrenal hormones depleted hepatic clock gene rhythms [44]. Nutrients and food factors can also modulate cellular circadian clocks or clock systems throughout the body. Thus, high-fat diet (HFD) consumption leads to increased food ingestion during the light period in mice, which may contribute to alterations in body weight regulation and to altered circadian patterns of circulating metabolic markers such as leptin and insulin, clock-regulating nuclear factors RORα and PPARα, hypothalamic neuropeptides AgRP and NPY, and factors involved in lipid metabolism [45]. In addition, HFD consumption has been reported to alter AMPK kinase signaling, which is activated in situations of energy shortage, leading to aberrant clock gene expression in comparison with the expression associated with a normal diet [46]. This HFD-mediated AMPK signaling alteration was improved in mice with time-restricted feeding despite equivalent caloric consumption as those with ad libitum access, leading to protection against obesity, hyperinsulinemia, hepatic steatosis, and inflammation [47,48]. In addition, HFD consumption in mice under constant darkness also alters the circadian locomotive rhythms, increasing their locomotive activity [45]. In humans, changes in dietary carbohydrate and fat percentage, from 55% carbohydrate–30% fat to 40% carbohydrate–45% fat, led to alterations in cortisol circadian oscillation, inflammatory and metabolic gene expression profiles, and *PER* gene expression rhythms in monocytes [49]. This study highlights the importance of not only the type of diet but also the proportion of its constituents. High salt (HS) diets have also been shown to affect biological rhythms. Thus, HS feeding showed alterations in intrarenal circadian rhythmicity with a significant delay in the peak expression of *Bmal1* and *Cry1* and *Per2* expression suppression in the renal inner medulla [50].

Recently, the gut microbiota has emerged as a key factor in metabolic modulation, and its potential influence on circadian rhythms is critical because it regulates the energy derived from food and modulates the levels of host- and diet-derived products [51]. Thus, changes in the gut microbiota induced by diet can affect the gut clock, influencing the organism’s homeostasis. Indeed, gut microbiota composition undergoes circadian oscillations in both mice and humans [51,52]. The microbiota also seems to influence several neuronal functions, and therefore, it may be possible that the central clock or different brain areas receive “nutritional” information in a cyclic manner through gut microbiota interplay [51,53,54].

Regarding individual nutrients contained in foods, the potential effect of some amino acid residues on sleep/wake cycle alterations has been evaluated. Thus, the consumption of 3 g L-serine 30 min before bedtime combined with bright-light exposure during the morning has been shown to be useful for enhancing light-induced phase resetting in humans [55]. Moreover, it is known that tryptophan is involved in sleep regulation since it is a precursor of serotonin and melatonin, hormones involved in sleep latency and quality, respectively [56]. Thus, the consumption of tryptophan-enriched cereals (60 mg tryptophan twice a day in the morning and at night) has been shown to increase sleep efficiency, actual sleep time, and immobile time in elderly people aged 55–75 years old [56]. Moreover, the consumption of cereals enriched with tryptophan, adenosine-5′-phosphate, and uridine-5′-phosphate at night increased sleep efficiency in infants 8–16 months old with sleep problems [57]. In addition, a diet rich in cherries or cherry juice, which contain a high concentration of tryptophan, serotonin, and melatonin, can also produce beneficial effects on sleep/wake rhythms in both middle-aged and elderly people, for example, reducing insomnia or increasing sleep time and efficiency [58,59,60,61]. However, their beneficial effects are dependent on the type of cherry cultivar [58]. In addition to tryptophan, other compounds contained in cherries could be involved in their effects, such as phytomelatonin, the original melatonin of plants, or polyphenols [59,60]. Moreover, fruits rich in serotonin may also help with sleep problems. Thus, the consumption of 2 kiwis 1 h before bed for 4 weeks has been shown to improve sleep quality in adults with sleep disturbances [62].

### 2.3. Xenohormesis

In addition to nutrients, heterotrophs are able to sense chemical cues mediated by non-nutrients synthesized by plants under adverse conditions, also called phytochemicals, promoting the adaptative capacity of these organisms. This process is known as xenohormesis [63,64]. In fact, some of these phytochemicals, such as polyphenols, have been associated with the prevention and/or treatment of chronic diseases, such as hypertension, cancer, diabetes, obesity, and other medical conditions [65].

## 3. Phenolic Compounds: Eating Patterns and Diseases

Regarding xenohormesis, one of the most important groups of phytochemicals is composed of phenolic compounds. These are secondary metabolites produced by plants in response to several types of stress: drought, high or low temperatures, microbial infections or consumption by herbivores, among others [66,67,68,69,70].

There are thousands of phenolic compounds described in plants that have the presence of at least one phenolic ring in common (more than 8000 described structures) [71]. These compounds can be divided into two main groups based on their chemical structure: flavonoids and nonflavonoids. Flavonoids are the most widely distributed compounds [72].

Figure S1 shows the main classes of phenolic compounds within these two categories. In general, these compounds can be found in plants as polymeric forms and/or linked to sugars [73]. For example, high-molecular-weight condensed tannins, also known as proanthocyanidins, are polymers composed of repetitions of flavanol units [74].

There are several health benefits derived from the consumption of fruits and vegetables rich in phenolic compounds [72,75]. Furthermore, these compounds confer bitterness, astringency, flavor, color, and oxidative stability to fruits and vegetables. In this sense, polyphenols are primarily responsible for the red colors and bitterness of wine.

### 3.1. Phenolic Contents in Vegetable Products

Fruits, cocoa products, and beverages, such as tea and wine, are the main sources of phenolic compounds in the human diet [72,76]. These compounds are located throughout the plant, including the roots, leaves, and fruits. Thus, leaves and stems contain higher amounts of these compounds, primarily in their monomeric form, while polymeric polyphenols are present in the vacuoles, leaves, epidermis, flowers, and fruits.

Table S1 shows the total polyphenol contents determined by the Folin–Ciocalteau assay and obtained from the Phenol-Explorer database [77,78,79]. Among the fruits and vegetables included in Table S1, cocoa, beans, and walnuts had the highest polyphenol content (5624.23, 1234.38, and 1574.82 mg/100 g FW, respectively). Other sources rich in phenolic compounds are pomegranates, plums, strawberries, and oranges, whose polyphenol contents range from approximately 400 to 280 mg/100 g FW. It must be taken into account that these values are the average of few reported independent studies, so these values can differ due to several factors.

The profile of phenolic compounds present in plants differs among species. In this regard, citrus fruits are rich in flavanones and flavones such as naringenine and hesperidin [80], while high-colored fruits such as cherries, grapes, and berries are rich in anthocyanins such as cyanidin and malvidin [81]; additionally, beverages such as wine, coffee, and tea contain high amounts of flavanols such as epigallocatechin gallate, catechin, and epicatechin [82]. These last compounds are usually found in vacuolar juices and in the epidermis [72,83]. Among the nonflavonoids, lignans (e.g., pinoresinol and matairesinol) are widespread in seeds and nuts [83].

### 3.2. Factors Affecting Polyphenol Composition

As previously explained, several factors can affect the content of phenolic compounds within the same species. In fact, both agricultural and technological factors have been shown to influence the phenolic profiles of plants. Within these two groups, variety, environment, soil fertilization, irrigation system, and ripening stage are the main agricultural factors. On the other hand, postharvest treatments arise within the technological factors.

Among all the factors, variety is one of the most important because it strongly conditions the profile of phenolic compounds. For example, in the literature, more than 7500 different apple varieties are described [84], and as Kalinowska et al. describe [85], the content of their phenolic compounds varies deeply. For instance, the total phenolic content can range from 56 mg GAE/100 g FW in Gala apples to 221 mg GAE/100 g FW in Panaia apples [86]. Interestingly, these two apples are red varieties. Francini and Sebastiani (2013) suggest that the genetic variability between different apples has an important impact on the phenolic profile [87]. Some of these changes can be explained by the expression of genes involved in the biosynthetic pathway of phenolic compounds, which differ between varieties. For example, in grapes, a lack of expression of the anthocyanidin 3-*O*-glucosyltransferase 2 (UFGT) gene, an enzyme crucial for the synthesis of anthocyanins [88], is observed in the white varieties compared to the red varieties.

Environmental factors also modulate the phenolic composition of fruits and vegetables. For instance, water availability, temperature, light exposure, and soil salt are described as important modulators of phenolic synthesis [66,67,68,69,70]. In fact, fruits from the same variety cultivated in different areas present different contents of phenolic compounds [89]. For example, Häkkinen and Törrönen observed significant differences in the total phenolic contents in berries cultivated in Finland (49.3 mg/100 g FW) compared to those cultivated in Poland (36.1 mg/100 g FW) [89]. These results highlight the importance of environmental factors in the content of phenolic compounds in plants.

Finally, regarding agronomical factors, controversial results have been observed in organic cultivation concerning phenolic content. Hence, some studies have shown that this agricultural practice increases the amount of phenols in the cultivar. For example, Stracke et al. showed that “Golden Delicious” apples from organic cultivars had 14%–19% greater phenolic content than apples from nonorganic cultivars [90]. However, in the study of Winter et al. [91], the authors concluded that inconsistent differences exist between organic and conventional cultivars. In another study, Mulero et al. [92] described that although significant differences were observed between unripe organic and nonorganic grape cultivars, these differences disappeared when grapes reached the ripening stage. Therefore, more research is needed in this field.

Technological factors include all post-harvesting treatments, such as cleaning, storage, antimicrobial treatments, and minimal processing. In this sense, the mixture of gasses used during storage can modulate the synthesis of phenolic compounds. Moreover, wounding of plants during harvesting induces the production of phenolic compounds as a mechanism of defense [93].

Taking into consideration all these facts, it is evident that the profiles of phenolic compounds of fruits and vegetables are highly variable. Therefore, it is plausible to consider that local fruits cultivated in specific areas and following determined agricultural practices will have a specific profile of phenolic compounds. In relation to the xenohormesis theory, the external signals conferred by these plant polyphenols may prepare the body for the local upcoming processes. All this may reinforce the consumption of local products because it may provide some benefits to the organism compared to other fruits.

### 3.3. Polyphenols and Diseases

As shown in Table S1, the beneficial effects of fruits and vegetables rich in phenolic compounds are wide, and these phytochemicals exert anti-inflammatory, antioxidant, anticarcinogenic, cardioprotective, and antihypertensive activities, among others. The mechanisms underlying these effects are based on the capacity of these compounds to modulate basic biochemical pathways related to inflammation and lipid and energy metabolism [94]. Moreover, these compounds can exert their action through epigenetic mechanisms. For instance, these compounds have been observed to regulate miRNA expression [95].

The health effects of fruit and vegetable consumption are partially attributed to specific polyphenols in each plant that can act as bioactive compounds. For example, the flavanols (+)-catechin and (-)-epicatechin are some of the main bioactives present in very common fruits, such as apples, cocoa, and grapes [72,82,94,96,97,98,99,100,101,102,103,104,105,106,107,108]. However, in citrus fruits such as oranges and lemons, these activities are related to the flavanones hesperidin and naringenin [72,97,109,110,111]. It is important to highlight that the health effects of polyphenols are mainly attributed to the product of their metabolism. In this sense, polyphenols are extensively conjugated in the small intestine and the liver by specific enzymes to generate glucuronide, sulfate, and methylated derivatives. Most of the polyphenols are not absorbed in the small intestine and reach the colon, where they are subjected to the activity of the gut microbiota [112,113]. Therefore, in addition to the agricultural and technological factors that influence the contents of polyphenols in plants, host internal factors such as the microbiota or enzyme activities may also have an impact on the effects of these compounds.

### 3.4. Eating Patterns

Traditionally, fruits and vegetables were produced and consumed during a limited season due to the climatic conditions of each cultivar. For example, the ripening of cherries and strawberries was limited to spring, whereas orange ripening limited the production to autumn and winter. However, currently, these patterns are changing because of the globalization that allows for the consumption of vegetable and fruit products throughout the year. For example, Spain is the major producer of oranges in the European Union (EU), producing an estimated 55% of oranges. However, this production is limited to the autumn and winter seasons. Thus, during the rest of the year, oranges in the EU are imported from countries in the south hemisphere, such as South Africa, Argentina, and Uruguay [114]. This allows for the maintenance of this product in the markets throughout the year. In addition, the production of fruits and vegetables in greenhouses also contributes to avoiding the seasonality of some products. For example, tomatoes and cucumbers can be found in the market all year due to this agricultural practice [115]. However, it must be taken into account that some of these fruits and vegetables that are imported are harvested before reaching maturity (climacteric fruits). Moreover, those that are produced in greenhouses undergo different postharvesting treatments than fruits traditionally cultivated. Thus, considering that these are important factors affecting phenolic synthesis, the contents of phenolic compounds can change, and consequently, this may also modify the health effects derived from their consumption.

## 4. Mechanisms Implicated in the Modulation of Metabolism by Seasonal Consumption of Polyphenol-Rich Fruits

**Figure 3:**
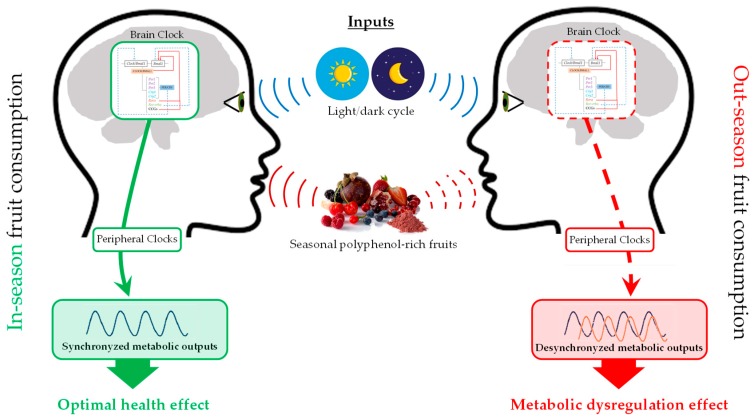
Interaction between gene regulation of biological rhythms, seasonal variation of plant polyphenols composition, and health seasonal effects.

As explained above, taking into account that each plant contains a distinctive composition of (poly)phenols based on the environment in which they were harvested, it is plausible to believe that seasonal consumption of polyphenol-rich fruits could induce marked changes in the regulation of physiology and metabolism depending on when they are consumed. Actually, it has been confirmed that both circannual and circadian rhythms influence the health outcomes of polyphenols. Figure 3 schematizes the interaction between gene regulation of biological rhythms, seasonal variation of plant polyphenols composition, and health seasonal effects. However, there is still a lack of information about this fact, and scarce studies can be found in the literature. Table 1 shows a compilation of dietary interventions and their health effects modulated by circadian and circannual rhythms.

### 4.1. Circannual Rhythms

In this sense, recent studies performed in dietary-induced obese F344 rats demonstrated that the consumption of sweet cherry *Prunus avium* L. for ten weeks exerted a marked deleterious photoperiod-dependent effect, increasing whole-body fat oxidation and circulating levels of glucose and insulin when it was consumed out of season [116,117]. These effects were partially explained by a downregulation of the phosphorylation levels of the downstream postreceptor target of insulin, Akt2, and an enhancement of fatty acid transport and β-oxidation-related pathways in skeletal muscle [116]. In addition, the consumption of *Prunus avium* L. out of season changed the morphology of white adipose tissue, increasing cell area and decreasing the number of adipocytes, which was mainly attributed to the downregulation of the expression of key genes involved in adipose tissue fat metabolism [117]. Interestingly, in brown adipose tissue, the fatty acid transporter *Cd36* was also downregulated, suggesting a lower capacity of brown adipocytes to absorb and oxidize fat (less thermogenic activity) [117]. Finally, in the central nervous system, the consumption of *Prunus avium* L. also modulated the hypothalamic leptin system regulating *Agrp* and *Ptp1B* mRNA levels only when it was consumed out of season [118].

Similarly, the out-of-season consumption of navelina oranges (*Citrus x sinsensis*) for ten weeks in the same animal model also exerted deleterious effects induced by an obesogenic diet, such as dyslipidemia and insulin resistance, increasing fatty acid synthesis in white adipose tissue and downregulating lipid uptake and β-oxidation in brown adipose tissue [119].

Relevantly, several epidemiological studies have shown how biological rhythm misalignment can contribute to a wide variety of metabolic disorders, including obesity, dyslipidemia, insulin resistance, and hypertension [120]. Therefore, the physiological and molecular changes elicited by seasonal fruit consumption could be partially explained by the modulation of the mammalian clock system. In fact, the consumption of both *Prunus avium* L. and *Citrus x sinsensis* has been correlated with the modulation of mRNA levels of different peripheral clock-related genes such as *Nr1d1* in skeletal muscle and *Per2* and *Cry1* in the liver and white adipose tissue of healthy rats when they are consumed out of season [116,117,119]. In accordance, previous studies performed by our group have demonstrated that grape seed proanthocyanidins (PAs), a subclass of flavonoids with a marked protective effect against diet-induced dyslipidemia and insulin resistance [94], could significantly modulate both central and peripheral biological rhythms in male Wistar rats. Specifically, an acute dose of 250 mg/kg body weight of PAs maintained elevated melatonin levels (nocturnal) at the beginning of the light phase and altered the rhythmic oscillations of some important circulating metabolites in healthy rats [121]. This phenotypic alteration was concomitant with the regulation of the hypothalamic expression pattern of clock genes such as *Bmal1* and *Nampt* [121]. In addition, different physiological doses of PAs for four weeks in diet-induced obese rats also modulated the levels of peripheral clock components such as *Bmal1, Nampt, Sirt1*, and NAD^+^ in a positive dose-dependent manner in the liver, gut, and white adipose tissue of these animals [8,122,123], suggesting that PAs could modulate physiology and metabolism by adjusting the peripheral and central molecular clock system in the obese state.

### 4.2. Circadian Rhythms

Several other plant (poly)phenols have been described to affect the central and peripheral clock systems. Epigallocatechin-3-gallate (EGCG), the major catechin found in green tea (*Camellia sinensis*), has been demonstrated to possess beneficial effects on obesity-related parameters, including decreasing body weight, reducing cholesterol and triglyceride levels in the liver and plasma, and improving glucose homeostasis [128]. Notably, in the search for the mechanisms of action of EGCG, Mi et al. [126] recently demonstrated for the first time that EGCG may ameliorate these obesity-related metabolic alterations by regulating the rhythmic expression of the circadian clock genes in C57BL/6J mice (Table 1). Specifically, EGCG partially normalized the circadian expression levels of the clock genes, such as *Clock*, *Bmal1*, and *Cry1,* by regulating the levels of *Sirt1* and *PGC1α* in both the liver and white adipose tissue. In addition, resveratrol, another body fat-lowering polyphenol found in grapes and red wine, restored the circadian desynchrony of lipid metabolism induced by high-fat-diet modification of the rhythmic expression of both clock genes (*Clock*, *Bmal1*, *Per2*, and *Rev-Erbα*) and clock-controlled lipogenic genes (*Sirt1*, *Pparα*, *Srebp-1c*, *Acc1*, and *Fas*) in white adipose tissue [129,130]. Cichoric acid, a polyphenol component from *Echinacea purpurea* that exhibits preventive effects against fatty liver disease, has also demonstrated *Bmal1* resistance to fatty liver accumulation by enhancing the *Akt/GSK3β* signaling pathways and modulating the downstream gene expressions involved in lipid metabolism [131]. Finally, cinnamic acid, another phenolic acid abundant in fruits that has attracted attention in recent years due to its antioxidant and antidiabetic properties [132], has been reported to shorten the circadian period of the molecular clock in differentiated neuronal cells as well as to reduce the free-running period of behavioral rhythms in mice [133].

All these studies might provide valuable data to design strategies for polyphenol-rich fruit consumption to counteract the metabolic alterations related to obesity by restoring the biological rhythm desynchrony induced by obesogenic behavior and ultimately normalizing the rhythmic expression of most of the important metabolic-related transcripts and metabolites. However, further comprehensive investigations are required to elucidate the underlying mechanisms through which (poly)phenols modulate the central and peripheral clock machinery.

## 5. Conclusions

The bidirectional interaction between phenolic compounds and biological rhythms strongly affects the beneficial effects derived from their consumption. In this sense, the different factors that modulate the synthesis of these compounds in plants are essential when evaluating the impact of this type of plant product on health. Different changes in metabolism may be observed in humans based on the consumption of seasonal products due to the particular phenolic profile that the product contains. This fact highlights the need for more studies focused on the impact of these specific phenolic profiles on health that would allow us to define more precise dietary recommendations regarding fruits and vegetables.

## Figures and Tables

**Table 1:** Dietary interventions and their health outcomes modulated by circadian and circannual rhythms.

Dietary Intervention	Experimental Model	Health Outcomes	Time Scale		References
			Circadian Rhythms ^a^	Circannual Rhythms ^b^	
***Human models***					
Catechin-rich green tea	Healthy young men	Reduced postprandial plasma glucose concentration	Evening (17:00 h)	n.a.	[124]
Polyphenol-Rich Grape-Wine Extract	Mildly hypertensive males and females	Lowered ambulatory systolic and diastolic blood pressure	Day-time	n.a.	[125]
***Animal models***					
Epigallocatechin-3-gallate	C57BL/6J mice	Ameliorated diet-induced metabolic misalignment by regulating the rhythmic expression of the circadian clock genes in the liver and fat adipose tissue	Night-time	n.a.	[126]
Grape seed proanthocyanidin extract	Male Wistar rats	Modulated the plasma melatonin level	Day-time	n.a.	[121]
Resveratrol	Male Wistar rats	Antioxidant	Night-time	n.a.	[127]
		Pro-oxidant	Day-time	n.a.	[127]
Red grapes (Traditional consumption: L6)	Standard (STD)-fed and cafeteria (CAF)-fed male Fischer 344 rats	Increased hypothalamic leptin sensitivity	n.a.	L6	[118]
Sweet cherries (Traditional consumption: L18)	STD-fed Fischer 344 male rats	Decreased blood nonesterified free fatty acids (NEFAs)	n.a.	L18	[116]
		Increased activation of fatty acid transport, β-oxidation-related pathways, and circulating glucose and insulin levels	n.a.	L6	[116]
	CAF-fed male Fischer 344 rats	Enhanced detrimental impact of CAF diet related to glucose metabolism.	n.a.	L6	[116]
	STD-fed and CAF-fed male Fischer 344 rats	Increased hypothalamic leptin sensitivity	n.a.	L6	[118]

^a^ Daytime (light cycle); night-time (dark cycle). ^b^ L6: Short-day photoperiod (6 h light/day); L18: long-day photoperiod (18 h light/day). n.a. information not available.

## Supplementary Materials

The following are available online at https://www.mdpi.com/2072-6643/11/11/2602/s1, Figure S1: Basic chemical structures of the main classes of phenolic compounds. According to their structure, phenolic compounds can be classified into two major families: flavonoids and nonflavonoids, and they can be further divided into several subclasses, Table S1: Total phenolic contents of relevant plant products, their most important bioactive phenols, and their health benefits.
