# Supplementary material for: Chrononutrition and Polyphenols: Roles and Diseases
Source: Nutrients. 2019 Oct 30;11(11):2602. doi: 10.3390/nu11112602 (PMC6893786; doi:10.3390/nu11112602)
Supplement: Supplementary file 1 [file nutrients-11-02602-s001.pdf]

nutrients

Review

Chrononutrition and Polyphenols: Roles and Diseases

Anna Arola-Arnal [1], Álvaro Cruz-Carrión [1], Cristina Torres-Fuentes [1], Javier Ávila-Román [1], Gerard Aragonès [1], Miquel Mulero [1], Francisca Isabel Bravo [1], Begoña Muguerza [1], Lluís Arola [1,2] and Manuel Suárez [1,*]

1 Nutrigenomics Research Group, Departament de Bioquímica i Biotecnología, Universitat Rovira i Virgili, 43007 Tarragona, Spain; anna.arola@urv.cat (A.A.-A.); alvarojavier.cruz@urv.cat (A.C.-C.); cristina.torres@urv.cat (C.T.-F.); franciscojavier.avila@urv.cat (J.Á.-R.); gerard.aragones@urv.cat (G.A.); miquel.mulero@urv.cat (M.M.); franciscaisabel.bravo@urv.cat (F.I.B.); begona.muguerza@urv.cat (B.M.); lluis.arola@urv.cat (L.A.)
2 Technological Unit of Nutrition and Health, EURECAT-Technology Centre of Catalonia, 43204 Reus, Spain
* Correspondence: manuel.suarez@urv.cat (M.S.); Tel.: +34-977-558-630

Received: date; Accepted: date; Published: date

Supplementary Materials: The following are available online at www.mdpi.com/xxx/s1, Figure S1: Basic chemical structures of the main classes of phenolic compounds. According to their structure, phenolic compounds can be classified into two major families: flavonoids and nonflavonoids, and they can be further divided into several subclasses, Table S1: Total phenolic contents of relevant plant products, their most important bioactive phenols and their health benefits.

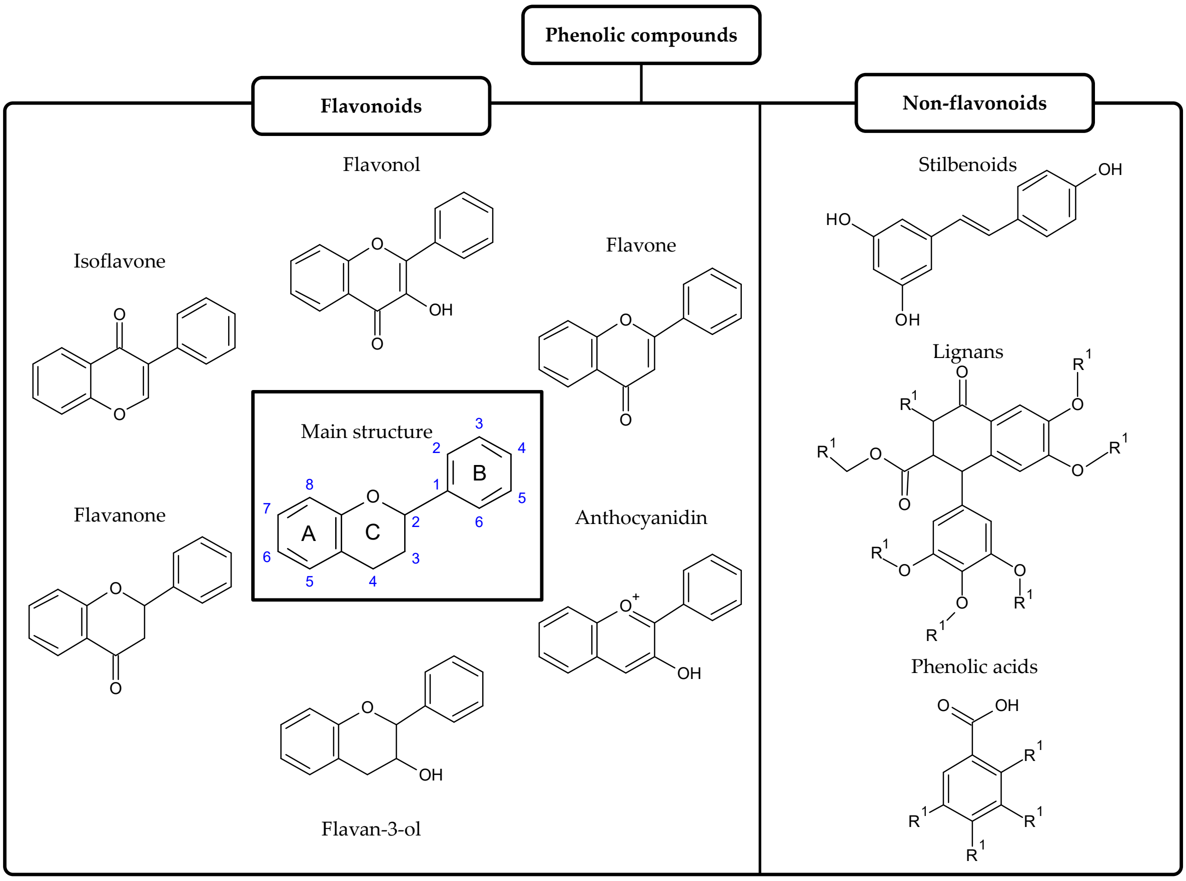

Figure S1. Basic chemical structures of the main classes of phenolic compounds. According to their structure, phenolic compounds can be classified into two major families: flavonoids and nonflavonoids, and they can be further divided into several subclasses.

Table S1. Total phenolic contents of relevant plant products, their most important bioactive phenols and their health benefits.

Plant product	Scientific name	Total content of phenolic compounds (mg/100 g FW)*	Most important bioactive phenols	Benefits to human health	References
Apple (whole, raw)	*Malus domestica*	200.96	(+)-Catechin<br>(-)-Epicatechin<br>Quercetin<br>Kaempferol	Anti-ulcer,<br>Anti-allergy,<br>Antioxidant,<br>Antihypertensive,<br>Anticancer,<br>Antidiabetic,<br>Anti-inflammatory,<br>Cardioprotective	[1–8]
Apricot (raw)	*Prunus armeniaca L.*	133.00	Quercetin<br>Kaempferol	Antioxidant,<br>Anticancer,<br>Antidiabetic,<br>Anti-inflammatory	[4–8]
Broccoli (raw)	*Brassica oleracea var. italica*	198.55	Quercetin<br>Myricetin	Anticancer	[9]
Cocoa, powder	*Theobroma cacao*	5624.23	(+)-Catechin<br>(-)-Epicatechin	Antioxidant,<br>Antihypertensive,<br>Antidiabetic,<br>Anti-inflammatory	[3,4]
Coffee (beverage, filtered)	*Coffea arabica L.*	266.70 mg/100 mL	Chlorogenic acid<br>Caffeic acid<br>*p*-Coumaric acid	Antioxidant,<br>Anti-inflammatory,<br>Cardioprotective	[10–14]
Common beans (whole)	*Phaseolus vulgaris L.*	1234.38	(+)-Catechin<br>Quercetin<br>Vanillin acid<br>Ellagic acid<br>Caffeic acid<br>Ferulic acid<br>Gallic acid<br>Chlorogenic acid<br>Sinapic acid	Antidiabetic,<br>Anti-obesity,<br>Antioxidant,<br>Anti-inflammatory,<br>Chemoprotective	[15]
Grape (black)	*Vitis vinifera L*	184.97	(+)-Catechin<br>(-)-Epicatechin<br>Quercetin<br>Kaempferol<br>Chlorogenic acid<br>Caffeic acid<br>*p*-Coumaric acid<br>Resveratrol<br>Proanthocyanidins	Anti-ulcer,<br>Anti-allergy,<br>Antiproliferative,<br>Antioxidant,<br>Antihypertensive,<br>Antidiabetic,<br>Anti-inflammatory,<br>Anticancer,<br>Anti-obesity,<br>Cardioprotective	[1,2,12–14,16–18,3–8,10,11]

Lemon	*Citrus limon*	59.80	Hesperidin<br>Naringenin	Antioxidant,<br>Anti-inflammatory,<br>Anticancer,<br>Cardioprotective	[4,8,19–21]
Mango	*Mangifera indica L.*	144.77	Mangiferin	Antioxidant,<br>Anticancer,<br>Antidiabetic,<br>Anti-allergic,<br>Anti-obesity	[22,23]
Nectarine (whole)	*Prunus persica var. nucipersica*	55.44	Chlorogenic acid<br>Caffeic acid<br>*p*-Coumaric acid	Antioxidant,<br>Anti-inflammatory,<br>Cardioprotective	[10–14]
Olive (green, raw)	*Olea europaea L.*	161.24	Tyrosol<br>Hydroxytyrosol	Anticancer,<br>Anti-inflammatory,<br>Antioxidant,<br>Cardioprotective	[24–28]
Onion (red, raw)	*Allium cepa L. var. cepa*	102.83	Quercetin<br>Kaempferol	Anti-ulcer,<br>Anti-allergy,<br>Antiproliferative,<br>Antioxidant,<br>Anticancer,<br>Antidiabetic,<br>Anti-inflammatory,<br>Cardioprotective	[2,4–8,24]
Orange (blond)	*Citrus sinensis L.*	278.59	Hesperidin<br>Naringenin	Antioxidant,<br>Anti-inflammatory,<br>Anticancer,<br>Cardioprotective	[4,8,19–21]
Parsley (fresh)	*Petroselinum crispum Mill.*	89.27	Apigenin	Antioxidant,<br>Anticancer,<br>Anti-inflammatory,<br>Cardioprotective	[4,29,30]
Peach (whole)	*Prunus persica L.*	279.08	Chlorogenic acid<br>Caffeic acid<br>*p*-Coumaric acid	Antioxidant,<br>Anti-inflammatory,<br>Cardioprotective	[10–14]
Pear (whole)	*Pyrus communis*	107.91	(+)-Catechin<br>Chlorogenic acids	Antioxidant	[2]
Pomegranate (peel, juice, seed)	*Punica granatum L.*	410.03[a]	Anthocyanidins	Antioxidant,<br>Cardioprotective	[31,32]
Plum (fresh)	*Prunus domestica L.*	409.79	Chlorogenic acids<br>Caffeic acid<br>*p*-Coumaric acid	Antioxidant<br>Anti-inflammatory<br>Cardioprotective	[10–14]
Raspberry (red, raw)	*Rubus idaeus L.*	154.65	Cyanidin<br>Malvidin<br>Gallic acid	Antioxidant,<br>Anti-obesity,<br>Anti-inflammatory,<br>Anticancer,<br>Antidiabetic,<br>Cardioprotective	[1,4,33–41]
Soybean (seed)	*Glycine Max L. Merr.*	354.00[b]	Genistein<br>Daidzin	Antioxidant,<br>Anticancer,<br>Antihyperlipidemic	[4,42–44]

Spinach (raw)	*Spinacia oleracea L.*	248.14	Chlorogenic acid<br>Caffeic acid<br>*p*-Coumaric acid	Antioxidant,<br>Anti-inflammatory,<br>Cardioprotective	[10–14]
Strawberry (raw)	*Fragaria L.*	289.20	Cyanidin<br>Malvidin<br>Gallic acid	Proapoptotic effects,<br>Antiproliferative,<br>Antioxidant,<br>Anti-obesity,<br>Anti-inflammatory,<br>Anticancer,<br>Antidiabetic,<br>Cardioprotective	[4,24,41,45,33–40]
Sweet cherry (raw)	*Prunus avium L.*	174.90	(+)-Catechin<br>Quercetin<br>Cyanidin<br>Malvidin<br>Chlorogenic acid<br>Caffeic acid<br>*p*-Coumaric acid	Antioxidant,<br>Anti-obesity,<br>Anti-inflammatory,<br>Cardioprotective	[2,4,10–14,34–36]
Tangerine	*Citrus tangerina*	192.00	Hesperetin<br>Naringenin	Antioxidant,<br>Anti-inflammatory,<br>Anticancer,<br>Cardioprotective	[4,8,19–21]
Tea (infusion)	*Camellia sinensis L. Kuntze*	104.48 (black)<br>61.86 (green)<br>mg/100 mL	Gallic acid<br>(+)-Catechin<br>(-)-Epicatechin	Antioxidant,<br>Anticancer,<br>Antidiabetic,<br>Antihypertensive,<br>Anti-inflammatory,<br>Cardioprotective	[3,4,33,37–41]
Walnut	*Juglans regia L.*	1574.82	Ellagic acid<br>Gallic acid	Antidiabetic,<br>Antioxidant	[46,47]

*Data from the Phenol-Explorer database [48–50]. [a][51]. [b][52]

References

1. Pandey, K.B.; Rizvi, S.I. Plant polyphenols as dietary antioxidants in human health and disease. *Oxid. Med. Cell. Longev.* **2009**, *2*, 270–278.

2. Rasouli, H.; Farzaei, M.H.; Khodarahmi, R. Polyphenols and their benefits: A review. *Int. J. Food Prop.* **2017**, *20*, 1700–1741.

3. Bladé, C.; Aragonès, G.; Arola-Arnal, A.; Muguerza, B.; Bravo, F.I.; Salvado, M.J.; Arola, L.; Suárez, M. Proanthocyanidins in health and disease. *BioFactors* **2016**, *42*, 5–12.

4. Del Rio, D.; Rodriguez-Mateos, A.; Spencer, J.P.E.; Tognolini, M.; Borges, G.; Crozier, A. Dietary (poly)phenolics in human health: structures, bioavailability, and evidence of protective effects against chronic diseases. *Antioxid. Redox Signal.* **2013**, *18*, 1818–1892.

5. Kawabata, K.; Mukai, R.; Ishisaka, A. Quercetin and related polyphenols: new insights and implications for their bioactivity and bioavailability. *Food Funct.* **2015**, *6*, 1399–1417.

6. Calderón-Montaño, J.M.; Burgos-Morón, E.; Pérez-Guerrero, C.; López-Lázaro, M. A Review on the Dietary Flavonoid Kaempferol | BenthamScience. *Mini Rev. Med. Chem.* **2011**, *11*, 298–344.

7. Dragovic-Uzelac, V.; Pospišil, J.; Levaj, B.; Delonga, K. The study of phenolic profiles of raw apricots and apples and their purees by HPLC for the evaluation of apricot nectars and jams authenticity. *Food Chem.* **2005**, *91*, 373–383.
8. Erlund, I. Review of the flavonoids quercetin, hesperetin, and naringenin. Dietary sources, bioactivities, bioavailability, and epidemiology. *Nutr. Res.* **2004**, *24*, 851–874.
9. Heneman, K.; Zidenberg-Cherr, S. Nutrition and Health Info Sheet: Flavonols. *Univ. Calif.* **2008**, 1–5.
10. Sato, Y.; Itagaki, S.; Kurokawa, T.; Ogura, J.; Kobayashi, M.; Hirano, T.; Sugawara, M.; Iseki, K. In vitro and in vivo antioxidant properties of chlorogenic acid and caffeic acid. *Int. J. Pharm.* **2011**, *403*, 136–138.
11. dos Santos, M.D.; Almeida, M.C.; Lopes, N.P.; de Souza, G.E.P. Evaluation of the Anti-inflammatory, Analgesic and Antipyretic Activities of the Natural Polyphenol Chlorogenic Acid. *Biol. Pharm. Bull.* **2006**, *29*, 2236–2240.
12. Clifford, M.N. Chlorogenic acids and other cinnamates – nature, occurrence, dietary burden, absorption and metabolism. *J. Sci. Food Agric.* **2000**, *80*, 1033–1043.
13. Möller, B.; Herrmann, K. Quinic acid esters of hydroxycinnamic acids in stone and pome fruit. *Phytochemistry* **1983**, *22*, 477–481.
14. Clifford, M.N.; Jaganath, I.B.; Ludwig, I.A.; Crozier, A. Chlorogenic acids and the acyl-quinic acids: discovery, biosynthesis, bioavailability and bioactivity. *Nat. Prod. Rep.* **2017**, *34*, 1391–1421.
15. Ganesan, K.; Xu, B. Polyphenol-Rich Dry Common Beans (Phaseolus vulgaris L.) and Their Health Benefits. *Int. J. Mol. Sci.* **2017**, *18*, 2331.
16. Savouret, J.F.; Quesne, M. Resveratrol and cancer: a review. *Biomed. Pharmacother.* **2002**, *56*, 84–87.
17. Smoliga, J.M.; Baur, J.A.; Hausenblas, H.A. Resveratrol and health--a comprehensive review of human clinical trials. *Mol. Nutr. Food Res.* **2011**, *55*, 1129–1141.
18. Nicoletti, I.; Bello, C.; De Rossi, A.; Corradini, D. Identification and quantification of phenolic compounds in grapes by HPLC-PDA-ESI-MS on a semimicro separation scale. *J. Agric. Food Chem.* **2008**, *56*, 8801–8808.
19. Roohbakhsh, A.; Parhiz, H.; Soltani, F.; Rezaee, R.; Iranshahi, M. Molecular mechanisms behind the biological effects of hesperidin and hesperetin for the prevention of cancer and cardiovascular diseases. *Life Sci.* **2015**, *124*, 64–74.
20. Tomás-Barberán, F.A.; Clifford, M.N. Flavanones, chalcones and dihydrochalcones – nature, occurrence and dietary burden. *J. Sci. Food Agric.* **2000**, *80*, 1073–1080.
21. Parhiz, H.; Roohbakhsh, A.; Soltani, F.; Rezaee, R.; Iranshahi, M. Antioxidant and anti-inflammatory properties of the citrus flavonoids hesperidin and hesperetin: an updated review of their molecular mechanisms and experimental models. *Phytother. Res.* **2015**, *29*, 323–331.
22. Masibo, M.; He, Q. Major Mango Polyphenols and Their Potential Human Health. *Compr. Rev. Food Sci. Food Saf.* **2008**, *7*, 309–319.
23. Imran, M.; Arshad, M.S.; Butt, M.S.; Kwon, J.H.; Arshad, M.U.; Sultan, M.T. Mangiferin: a natural miracle bioactive compound against lifestyle related disorders. *Lipids Health Dis.* **2017**, *16*, 1–17.

24. Renard, C.M.G.C. Extraction of bioactives from fruit and vegetables: State of the art and perspectives. *Lwt* **2018**, *93*, 390–395.

25. Vauzour, D.; Rodriguez-Mateos, A.; Corona, G.; Oruna-Concha, M.J.; Spencer, J.P.E. Polyphenols and human health: Prevention of disease and mechanisms of action. *Nutrients* **2010**, *2*, 1106–1131.

26. D'Archivio, M.; Filesi, C.; Di Benedetto, R.; Gargiulo, R.; Giovannini, C.; Masella, R. Polyphenols, dietary sources and bioavailability. *Ann. Ist. Super. Sanita* **2007**, *43*, 348–361.

27. Rigacci, S.; Stefani, M. Nutraceutical Properties of Olive Oil Polyphenols. An Itinerary from Cultured Cells through Animal Models to Humans. *Int. J. Mol. Sci.* **2016**, *17*, 843.

28. Martin-Pelaez, S.; Covas, M.I.; Fito, M.; Kusar, A.; Pravst, I. Health effects of olive oil polyphenols: recent advances and possibilities for the use of health claims. *Mol. Nutr. Food Res.* **2013**, *57*, 760–771.

29. Singh, M.; Kaur, M.; Silakari, O. Flavones: An important scaffold for medicinal chemistry. *Eur. J. Med. Chem.* **2014**, *84*, 206–239.

30. El Gharras, H. Polyphenols: Food sources, properties and applications - A review. *Int. J. Food Sci. Technol.* **2009**, *44*, 2512–2518.

31. Davidson, M.H.; Maki, K.C.; Dicklin, M.R.; Feinstein, S.B.; Witchger, M.S.; Bell, M.; McGuire, D.K.; Provost, J.C.; Liker, H.; Aviram, M. Effects of Consumption of Pomegranate Juice on Carotid Intima-Media Thickness in Men and Women at Moderate Risk for Coronary Heart Disease. *Am. J. Cardiol.* **2009**, *104*, 936–942.

32. Lansky, E.P. Pomegranate products useful in improving health and methods of use thereof 2002, *WO 02/0943*.

33. Gruz, J.; Novák, O.; Strnad, M. Rapid analysis of phenolic acids in beverages by UPLC-MS/MS. *Food Chem.* **2008**, *111*, 789–794.

34. Kong, J.M.; Chia, L.S.; Goh, N.K.; Chia, T.F.; Brouillard, R. Analysis and biological activities of anthocyanins. *Phytochemistry* **2003**, *64*, 923–933.

35. Prior, R.L.; Wu, X. Anthocyanins: Structural characteristics that result in unique metabolic patterns and biological activities. *Free Radic. Res.* **2006**, *40*, 1014–1028.

36. Kuntz, S.; Asseburg, H.; Dold, S.; Römpp, A.; Fröhling, B.; Kunz, C.; Rudloff, S. Inhibition of low-grade inflammation by anthocyanins from grape extract in an in vitro epithelial-endothelial co-culture model. *Food Funct.* **2015**, *6*, 1136–1149.

37. Jin, L.; Piao, Z.H.; Sun, S.; Liu, B.; Kim, G.R.; Seok, Y.M.; Lin, M.Q.; Ryu, Y.; Choi, S.Y.; Kee, H.J.; et al. Gallic Acid Reduces Blood Pressure and Attenuates Oxidative Stress and Cardiac Hypertrophy in Spontaneously Hypertensive Rats. *Sci. Rep.* **2017**, *7*, 1–14.

38. Hsu, C.L.; Yen, G.C. Effect of gallic acid on high fat diet-induced dyslipidaemia, hepatosteatosis and oxidative stress in rats. *Br. J. Nutr.* **2007**, *98*, 727–735.

39. Verma, S.; Singh, A.; Mishra, A. Gallic acid: Molecular rival of cancer. *Environ. Toxicol. Pharmacol.* **2013**, *35*, 473–485.

40. Punithavathi, V.R.; Prince, P.S.M.; Kumar, R.; Selvakumari, J. Antihyperglycaemic, antilipid peroxidative and antioxidant effects of gallic acid on streptozotocin induced diabetic Wistar rats. *Eur. J.*

Pharmacol. **2011**, *650*, 465–471.

41. Tomás-Barberán, F.A.; Clifford, M.N. Dietary hydroxybenzoic acid derivatives – nature, occurrence and dietary burden. *J. Sci. Food Agric.* **2000**, *80*, 1024–1032.
42. McCue, P.; Shetty, K. Health benefits of soy isoflavonoids and strategies for enhancement: A review. *Crit. Rev. Food Sci. Nutr.* **2004**, *44*, 361–367.
43. Tham, D.M.; Garner, C.D.; Haskell, W.L. Potential health benefits of dietary phytoestrogens: A review of clinical, epidemiological, and mechanistic evidence. *J. Clin. Endocrinol. Metab.* **2011**, *83*, 2223–2235.
44. Ganesan, K.; Xu, B. A critical review on polyphenols and health benefits of black soybeans. *Nutrients* **2017**, *9*, 1–17.
45. Pereira Lima, P.G.; Vianello, F.; Corrêa, C.R.; da Silva Campos, R.A.; Galhardo Borguini, M. Polyphenols in Fruits and Vegetables and Its Effect on Human Health. *Food Nutr. Sci.* **2014**, *5*, 1065–1082.
46. Vinson, J.A.; Cai, Y. Nuts, especially walnuts, have both antioxidant quantity and efficacy and exhibit significant potential health benefits. *Food Funct.* **2012**, *3*, 134–140.
47. Ma, Y.; Njike, V.Y.; Millet, J.; Dutta, S.; Doughty, K.; Treu, J.A.; Katz, D.L. Effects of walnut consumption on endothelial function in type 2 diabetic subjects. *Diabetes Care* **2010**, *33*, 227.
48. Rothwell, J.A.; Urpi-Sarda, M.; Boto-Ordonez, M.; Knox, C.; Llorach, R.; Eisner, R.; Cruz, J.; Neveu, V.; Wishart, D.; Manach, C.; et al. Phenol-Explorer 2.0: a major update of the Phenol-Explorer database integrating data on polyphenol metabolism and pharmacokinetics in humans and experimental animals. *Database (Oxford)* **2012**, *2012*, bas031.
49. Rothwell, J.A.; Perez-Jimenez, J.; Neveu, V.; Medina-Remón, A.; M'Hiri, N.; García-Lobato, P.; Manach, C.; Knox, C.; Eisner, R.; Wishart, D.S.; et al. Phenol-Explorer 3.0: a major update of the Phenol-Explorer database to incorporate data on the effects of food processing on polyphenol content. *Database* **2013**, *2013*, bat070.
50. Neveu, V.; Perez-Jiménez, J.; Vos, F.; Crespy, V.; du Chaffaut, L.; Mennen, L.; Knox, C.; Eisner, R.; Cruz, J.; Wishart, D.; et al. Phenol-Explorer: an online comprehensive database on polyphenol contents in foods. *Database (Oxford).* **2010**, *2010*, bap024.
51. Gözlekçi, S.; Saraçoglu, O.; Onursal, E.; Özgen, M.; Gozlekci, S.; Saracoglu, O.; Onursal, E.; Ozgen, M. Total phenolic distribution of juice, peel, and seed extracts of four pomegranate cultivars. *Pharmacogn. Mag.* **2011**, *7*, 161–164.
52. Nam, J.; Kang, S.; Hong, S.; Kim, S.; Jin, Y. Analysis of the Phenolic Content and Antioxidant Activities of Soybean Extracts from Different Regions and Cultivars. **2014**, *27*, 610–621.

© 2019 by the authors. Submitted for possible open access publication under the terms and conditions of the Creative Commons Attribution (CC BY) license (http://creativecommons.org/licenses/by/4.0/).
